# Correlated Inflammatory Responses and Neurodegeneration in Peptide-Injected Animal Models of Alzheimer's Disease

**DOI:** 10.1155/2014/923670

**Published:** 2014-04-13

**Authors:** James G. McLarnon

**Affiliations:** Department of Anesthesiology, Pharmacology and Therapeutics, University of British Columbia, Vancouver, BC, Canada V6T 1W3

## Abstract

Animal models of Alzheimer's disease (AD) which emphasize activation of microglia may have particular utility in correlating proinflammatory activity with neurodegeneration. This paper reviews injection of amyloid-**β** (A**β**) into rat brain as an alternative AD animal model to the use of transgenic animals. In particular, intrahippocampal injection of A*β*_1-42_ peptide demonstrates prominent microglial mobilization and activation accompanied by a significant loss of granule cell neurons. Furthermore, pharmacological inhibition of inflammatory reactivity is demonstrated by a broad spectrum of drugs with a common endpoint in conferring neuroprotection in peptide-injected animals. Peptide-injection models provide a focus on glial cell responses to direct peptide injection in rat brain and offer advantages in the study of the mechanisms underlying neuroinflammation in AD brain.

## 1. Introduction

Chronic inflammation is a characteristic feature of Alzheimer's disease (AD) brain [1, 2]. However, evidence supporting contributory roles of chronic inflammation as a causative factor in mediating neuronal damage in neurodegenerative diseases remains elusive. Indeed, a balanced perspective on roles of inflammation in AD suggests that cellular inflammatory responses can yield both beneficial and detrimental outcomes depending on a complexity of factors [3–5]. At present, the use of anti-inflammatory therapies for AD patients has demonstrated limited or minimal clinical utility. It is noteworthy that nonsteroidal anti-inflammatory drugs (NSAIDs) treatment, which primarily targets COX-2 enzymatic activity, has shown some efficacy in clinical studies [6–8]. The overall lack of benefits for anti-inflammatory-based therapies in AD may reflect the activation of multiple and complex signaling pathways mediating inflammatory reactivity.

A number of review articles are available which have considered the characteristic properties, including roles of inflammatory responses, demonstrated by a host of transgenic animal models [9–11]. In general, commonly used transgenic mouse models such as Tg2576 exhibit relatively subtle and modest degrees of chronic inflammation in the progression of disease [12]. In addition, transgenic animal models also do not generally manifest extensive amounts of neuronal damage and loss [13, 14]. A caveat, however, is that some transgenic models have been developed which exhibit significant neurodegeneration in aged mice [15–18]. A relevant point is that transgenic animal models are often not examined for neurodegeneration but instead more routinely tested for changes in cognitive performance in aged animals. One possible underlying reason for the benign levels of chronic inflammation in transgenic mouse AD models is the relatively slow and progressive accumulation in amyloid-*β* (A*β*) plaques in aged animals. It also can be considered that animal models using rats rather than mice may offer particular advantages in testing AD pathology due to closer similarities between the animal species and human tissue [19].

The injection of A*β* into rat brain serves as a stimulus for eliciting proinflammatory reactivity. Although the specific mechanisms by which A*β* deposition contributes to disease pathology are not well understood [20], deposits of peptide are noteworthy as chemotactic and activating stimuli for microglia, resident effector cells which mediate immune responses in brain [21, 22]. Considerable evidence has demonstrated that microglial activation serves as a critical transduction process contributing to inflammatory reactivity in AD brain. Once activated, microglial cells express and produce a plethora of inflammatory factors which in assemblage can be toxic to bystander cells including neurons. Results from* in vitro* studies have demonstrated that exposure of microglia to various forms of A*β* yields a milieu of inflammatory products with potential neurotoxic consequences [23–26].

## 2. Inflammatory Responses and Neuronal Damage/Impaired Behavior in Animals Receiving Injection of A*β*

Relatively few studies have examined effects of A*β* injection as a stimulus for inflammation-induced neurodegeneration in animals with early work focused on impairment in behavioral response. A number of criteria can be noted which reflect the validity in using peptide injection animal models. Firstly, A*β* should be injected at relatively low levels (low nM range) to approximate conditions in AD brain. The type of A*β* is also relevant since soluble species of peptide applied* in vivo* may be less effective compared with aggregated peptide [27, 28]. Secondly, peptide injection should be made into a well-defined brain region separate from the area of analysis for neuron viability. This procedure is required to minimize possible direct neuronal damage from the effects of injected peptide. The needle track can be used as a marker for specific placement of the injection system (see below). Thirdly, the extents of gliosis in response to peptide injection should be measured in proximity to neuronal expression to allow the possibility in correlating glial and neuronal responses.

In an early study, no significant behavioral impairment was reported in rats subjected to long-term hippocampal injection of A*β*_1-42_ [29]. Although concomitant neuronal viability was not measured, the lack of impaired behavior was suggested to reflect minimal loss of neurons in relevant areas of brain. The associated changes in microglial and astroglial activity were not determined in this work. A subsequent work concluded activation of glial cells underlay abnormal behavioral responses in rats receiving A*β*_1-42_ injected into the CA3 region of hippocampus [28]. The suggestion of glial responses as a factor in impaired behavior was based on findings of delayed impairment in behavior response which showed similar time course to gliosis. Another study reported increased astrogliosis and IL-1*β* immunoreactivity in microglia and neurons in animals receiving injections (into amygdala) of shorter length peptide, A*β*_25-35_ [30]. Although early histopathological effects of peptide injection were evident, the overall results indicated no severe impairment in behavior for injected animals.

A critical study using specific markers for microglia, astrocytes, and neurons was designed to examine roles of gliosis in animals receiving peptide injection [31]. Importantly, direct neuronal loss was determined in the work rather than recording abnormalities in behavior. Gliosis and viability of neurons were measured in rats injected with fibrillar A*β*_1-40_ (fAB) into striatum (forebrain area of CNS affected in AD). The results showed long-term effects (30 d after injection) of fAB were marked increases in microgliosis and astrogliosis with both cell types exhibiting enhanced iNOS expression. Although fAB induced considerable gliosis, differences in glial responses were noted in terms of spatial and morphological responses to peptide. Importantly, loss of striatal neurons was observed with fAB relative to controls (vehicle and soluble peptide) and correlated with glial responses. The authors concluded that inflammatory factors derived from peptide-stimulated glia could contribute to neuronal degeneration.

As noted above, some evidence suggests limited beneficial actions of NSAIDs in AD. One such compound, ibuprofen, was tested as an inhibitor of plaques in a transgenic animal model of AD [32]. Ibuprofen was found effective in inhibiting microglial activation and astrogliosis and plaque development. The positive effects of ibuprofen in the transgenic model prompted study of the compound in peptide-injected rat brain. Full-length A*β*_1-42_ was injected into the CA3 region of hippocampus and a battery of behavioral tests conducted after injection [33]. Behavioral abnormalities were measured after 30 d of peptide injection with ibuprofen administration found effective in restoring normal patterns of behavior. Furthermore, withdrawal of the compound from A*β*_1-42_-injected rats resulted in a progressive decline in behavior responses. Considerable astrogliosis accompanied long-term peptide injection with gliosis inhibited in animals administered ibuprofen. An increase in astrogliosis with ibuprofen withdrawal suggested the possibility of inflammatory response contribution to impaired behavior in the peptide injection animal model; however, concomitant effects of ibuprofen on microglial responses were not studied.

Inflammatory responses mediated by activated astrocytes and microglia were correlated with neuron damage following cerebroventricular infusion of A*β*_1-42_ in mouse brain [34]. This work also examined extents of gliosis and hippocampal levels of the proinflammatory factors IL-1*β*, TNF-*α*, and S100B. The administration of an aminopyridazine compound diminished numbers of activated glial cells and expression of a host of proinflammatory cytokines. Drug treatment was found effective in attenuating neuronal damage for CA1 neurons and maintaining levels of synaptophysin (synaptic vesicle protein). The authors concluded that inhibition of glial responses provided neuroprotection in the neuroinflammation animal model.

Recent work has examined behavioral performance in rats receiving bilateral intrahippocampal injection of aggregated A*β*_1-42_ [35]. Impaired learning and memory tasks were demonstrated in peptide-injected animals relative to controls. Oral administration of an anti-inflammatory pyrimidine derivative was found to improve behavioral responses over long-term (90 d) treatment periods. Interestingly, the pyrimidine compound demonstrated enhanced efficacy in protecting behavior compared to NSAID treatment. This work did not assess neuronal viability or microgliosis* in vivo* but reported enhanced astrogliosis following intrahippocampal A*β*_1-42_ injection which was reduced in animals receiving drug administeration. Accompanying* in vitro* experiments showed that A*β*_1-42_-induced macrophage (microglia) release of a host of proinflammatory factors was significantly reduced with administration of the pyrimidine compound. The overall results suggested inhibition of neuroinflammation as a mechanism for improving learning and memory in peptide-injected rat brain.

The mechanisms by which microglia (or astrocytes) respond to injected forms of A*β* in the animal model studies are not well understood. However, a diversity of cell receptors responsive to different forms of peptide have been implicated in mediating glial cellular responses. Putative receptors in microglia include scavenger receptor [36], scavenger receptor complex [37], formyl peptide receptor [38], and receptor for advanced glycation end products (RAGE) [39]. A complexity in transduction processes, including both calcium-dependent and -independent pathways, couples receptor activation to cellular functional responses. Products of activated microglia include superoxide [40], proinflammatory cytokines such as tumor necrosis factor-*α* [41] and interleukins IL-1*β* [42] and IL-6 [43] and excitatory amino acids including glutamate [44]. In essence an elevated milieu of inflammatory factors can be produced from A*β*-stimulated microglia resulting in localized brain microenvironments which are potentially toxic to bystander neurons. Thus an assemblage of inflammatory agents could act in concert to alter synaptic signaling and damage hippocampal and cortical neurons in the ongoing progression of AD pathology. It is important to note that activated microglia can also mediate anti-inflammatory activity, a point discussed below.

Work from this laboratory has systematically examined intrahippocampal injection of full-length peptide A*β*_1-42_ as an animal model of AD [45]. A focus of study is correlative changes between inflammatory responses and neuronal viability and the effects of pharmacological intervention in peptide-injected brain. Overall, a diversity of drugs including thalidomide [41], pyrazole compound 2-MBAPA [46], angiostatin [47], or antibody treatments including anti-VEGF [48] and anti-MAC-1 antibody for antigen CD11b [49] have been found efficacious in reducing microgliosis in the peptide-injection animal model of AD. The common aspect of drug action from this disparate group of compounds is generalized anti-inflammatory activity in the AD animal model. In all studies peptide-injected animals administered drugs or receiving antibody treatment demonstrated modest, but significant, increases in neuron viability.

We consider that the common finding in reduction of microgliosis could involve drug effects to reduce microglial chemotactic responses mediated by a host of chemokines and inhibition of subsequent cell activation mediated by a host of receptors (noted above). The finding that all treatment strategies lead to diminished microgliosis and associated increases in numbers of GCL neurons suggests correlation between inhibition of microgliosis and enhanced neuronal viability. It can be noted that, with the different drug administrations, levels of microglial immunoreactivity are still significantly higher compared to those observed in untreated control animals. In addition, astrogliosis is also increased in peptide-injected rat brain compared with controls. However, unlike microglial responses, astrogliosis appears minimally affected by any of the drug treatments noted above.

## 3. Practicalities and Experimental Example Using A*β*_1-42_ Intrahippocampal Injection as an AD Animal Model

### 3.1. Background

This model determines neuronal viability in the granule cell layer (GCL) and glial inflammatory responses (and changes in microvasculature) in the adjacent molecular layer (ML). The essential intent of the A*β*_1-42_ injection model is to initiate a chemotactic inflammatory response from resident microglia and astrocytes. A diversity of chemokines are upregulated in the animal model and implicated in enhancing microglial mobility to localize cells in proximity to amyloid deposition. Examples of increased chemokines include monocyte chemotactic protein, macrophage inflammatory peptide-1*α*, and interleukin IL-8. The chemotactic response to intrahippocampal injection of A*β*_1-42_ rapidly progresses to glial activation mediated by a host of cellular receptors (noted above) stimulated by peptide. In order to examine association of neuronal viability with inflammation, immunohistochemical staining with specific neuronal, microglial, and astrocytic markers is employed. Quantification of staining density can be done using specifically designed programs which measure the area density of cell markers. Typical cellular markers include NeuN (for neurons), Iba-1 (for microglia), and GFAP (for astrocytes). Changes in microvasculature expression can also be examined using antibodies for RECA-1 (rat endothelial cell antigen), laminin, or vWF (von Willebrand factor).

An important aspect of AD animal model study is to validate findings in comparison to results obtained in human AD brain (see below). In this case animal model data for specific cellular responses and functional processes can be utilized and compared with marker expression in samples of hippocampal and cortical human brain tissue obtained from nondemented (ND) controls and AD individuals. A number of brain banks serve critical roles for supply of human brain tissue.

### 3.2. Example of Data Recorded Using A*β*_1-42_ Injection Animal Model

In practice (see [46–49] for detailed methodology), stereotaxic injection of peptide (A*β*_1-42_ at 1 or 2 nM) is made into the CA1 region of rat hippocampus. The specific location of peptide injection is well-defined (established coordinates from bregma) by the needle track. Peptide injection evokes a prominent inflammatory response which is assessed at a distance from the injection site and located in the molecular layer (ML) of dentate gyrus. The viability of neurons is determined in the granule cell layer (GCL) adjacent to ML. The proximity in analytic regions allows assessment of correlative information between glial and neuronal responses. In particular extents of microgliosis, astrogliosis and neuronal loss can be determined. Furthermore changes in properties of microvasculature including abnormalities in the morphology of microcapillaries, changes in the density of microvessels, and increased leakiness of blood-brain barrier (BBB) can also be examined.

In effect the intrahippocampal injection of A*β*_1-42_ initiates an acute microglial response which transitions to a chronic inflammatory process within a short duration following peptide injection. The usual timeframe for analysis of inflammatory response is one week after injection. In practice, at 3 d following A*β*_1-42_ injection, microglial cells are localized in the vicinity of peptide and exhibit characteristic properties of an activated phenotype. Several controls are employed in the study including intrahippocampal injection of vehicle (phosphate buffer solution (PBS)) and reverse peptide (A*β*_42-1_), an inactive form of peptide. The former serves as a measure for quantification of the effects of peptide injection (A*β*_1-42_ versus PBS) and the latter as a measure of peptide activity (A*β*_1-42_ versus A*β*_42-1_). It can be noted that the glial, neuronal, and vasculature properties for the two controls exhibit very similar patterns of response.

**Figure 1:**
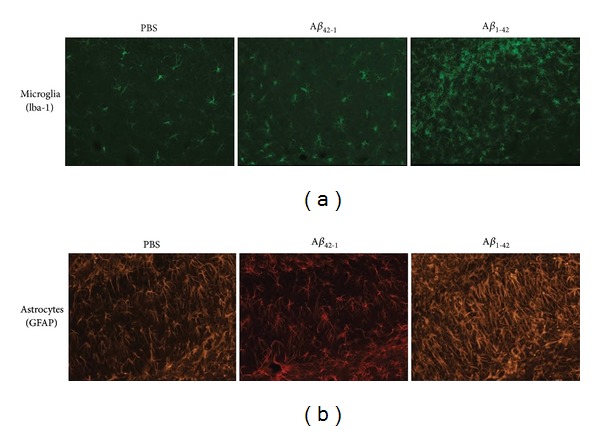
Glial responses in AD animal model. (a) Representative microgliosis (Iba-1 marker) for controls (PBS and reverse peptide A*β*_42-1_, respective left and middle panels) and A*β*_1-42_ (right panel). (b) Typical astrogliosis (GFAP marker) for the same animal groups as in (a).

Representative responses from the different animal groups treated in the peptide injection model are presented below (all data obtained at 7 d after peptide injection). Control animals receiving PBS injection exhibit low expression of Iba-1 indicating relatively small extents of microgliosis in ML (Figure 1(a), left panel). A similar pattern of staining is found in animals receiving injection of reverse peptide A*β*_42-1_ (Figure 1(a), middle panel). For both control animal groups, morphologies of cells appear ramified with elaborated cell processes suggesting cells may be in a quiescent and low level of activation. A very different profile of Iba-1 expression is found in rats injected with A*β*_1-42_. In this case a considerably enhanced level of microgliosis is observed (Figure 1(a), right panel). Previous work [49] has shown Iba-1 is increased fourfold in A*β*_1-42_ versus PBS-injected rat hippocampus (time point of 7 d after injection). Furthermore cells exhibit an altered morphology from controls including evidence for ameboid morphology (some retraction of processes and a trend to larger cell bodies). Examples for clustering of microglia, commonly involving a grouping of several cells, are also demonstrated in peptide-injected animals. Clustered and activated microglia, in proximity to deposits of peptide, are reported as a characteristic feature of AD inflammatory reactivity in animal models of AD [50, 51] and in AD brain tissue [52].

Representative patterns of astrocytic staining are presented in Figure 1(b). For controls, rats injected with either PBS or reverse peptide A*β*_42-1_ show modest levels of GFAP immunoreactivity (ir) in the ML of hippocampus (left and middle panels of Figure 1(b)). A considerably elevated GFAP expression is evident in animals injected with A*β*_1-42_ (right panel, Figure 1(b)). The increase in GFAP staining in peptide-injected rats indicates astrogliosis in the AD animal model. Previous work has demonstrated that GFAP ir is approximately doubled for A*β*_1-42_ versus PBS at a time point of 7 d after injection [41]. The morphology of astrocytes is similar between control and peptide-injected animals with cells showing extended processes.

**Figure 2:**
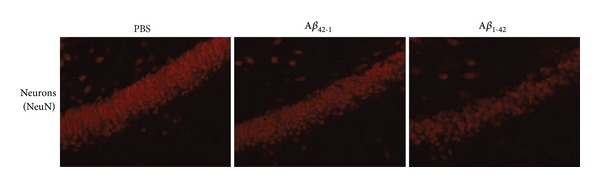
Viability of GCL neurons in animal model. Representative neuronal expression (NeuN marker) for the same animal groups as in Figure 1. Left and middle panels show NeuN for respective PBS and reverse peptide controls. Right panel is marker staining for A*β*_1-42_ injection.

The viability of neurons is evaluated in the GCL of dentate gyrus, adjacent to ML, using NeuN as a cell-type specific marker. Representative staining for granule cell neurons in control animals is shown in segments of GCL in Figure 2. PBS and reverse peptide-injected rats exhibit an intact GCL for NeuN expression; the results (Figure 2, left and middle panels) indicate that about 8 cells span the width of the GCL. Similar results are obtained using other markers for neuronal viability in control groups, for example, the protein calbindin [47]. An altered pattern of NeuN staining for rats injected with A*β*_1-42_ is demonstrated in Figure 2 (right panel) with GCL considerably reduced in width. The loss of viable neurons in GCL is a characteristic feature of the A*β*_1-42_-injected rat hippocampus.

**Figure 3:**
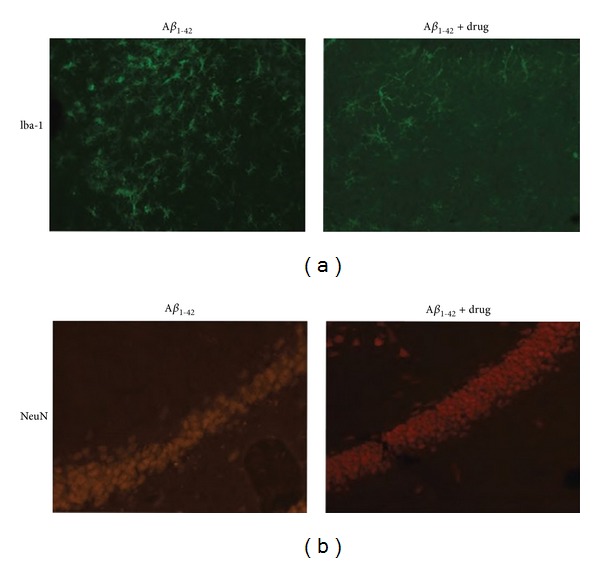
Microglial and neuronal responses in angiostatin-treated rats. (a) Representative microgliosis is shown for A*β*_1-42_-injected animals (left panel) and for peptide-injected animals receiving angiostatin treatment (right panel). (b) Typical expression of GCL neurons for untreated peptide-injected (left panel) and angiostatin-treated (right panel) animals.

This laboratory has used a diversity of animal treatments to target microgliosis in the peptide animal model. The underlying hypothesis is that inhibition of inflammatory reactivity could serve as a rationale strategy to confer neuroprotection. As an example, recent work has employed the antiangiogenic agent, angiostatin, as a novel putative anti-inflammatory factor [47]. Control animals (injected with vehicle, PBS) exhibit minimal extents of microgliosis (data not shown). Representative microgliosis is presented in animals receiving intrahippocampal injection of A*β*_1-42_ (Figure 3(a), left panel) and in peptide-injected animals receiving angiostatin (Figure 3(a), right panel). Angiostatin treatment of rats was associated with a marked reduction in levels of microgliosis. The patterns of Iba-1 immunostaining indicated a reduced cellular activation in drug-treated animals.

The corresponding neuronal staining in GCL layer is presented in Figure 3(b). Animals receiving the administration of angiostatin with A*β*_1-42_ injection exhibited increased numbers of GCL neurons compared with untreated A*β*_1-42_-injected animals. Indeed, the patterns of NeuN immunoreactivity and intactness of GCL were similar between peptide-injected rats receiving angiostatin and control animals (data not shown). The expression of neuronal viability, using calbindin as a marker, is also demonstrated for the different animal groups [47]. Thus angiostatin confers a modest, but significant, degree of neuroprotection in the AD animal model. The partial neuroprotection may reflect the abundance and complexity of inflammatory processes initiated by peptide injection. Interestingly, the extents of astrogliosis were not significantly reduced with angiostatin treatment of animals receiving A*β*_1-42_. A similar finding has been obtained in other studies [41, 49] whereby enhanced astrogliosis in peptide-injected rat hippocampus is not significantly modified by administration of anti-inflammatory compounds.

## 4. Establishing Animal Model Validity by Comparison with Data from AD Brain Tissue 

An important aspect in using animal models is to examine and test the validity of data in comparison to conditions in AD brain. The comparison can be facilitated by using cortical and hippocampal tissue isolated from controls (ND nondemented) and AD individuals. Specific guidelines are available to distinguish between ND and AD brain tissue including criteria based on extents of plaque deposition and tau formation [53, 54]. In our case human tissue is available from the Kinsmen laboratory brain bank located at the University of British Columbia. Independent analysis from trained pathologists has classified AD and ND cases and provided cortical and hippocampal tissue sections from the Kinsmen laboratory.

Our findings are consistent with previous work [2] in showing sections from AD brain tissue exhibit marked increases in microgliosis and astrogliosis compared with ND tissue [52, 55]. Overall, staining of gliosis activity in AD and ND tissue demonstrates similar patterns of immunoreactivity to those found between A*β*_1-42_ and PBS injection in the intrahippocampal A*β*_1-42_ injection animal model. Furthermore clusters of microglia in proximity to peptide are characteristic features in cortical and hippocampal sections isolated from human AD tissue [52]. A spectrum of similarities in morphological perturbations and abnormalities in properties of microvasculature are also evident between peptide-injected rat brain and tissue obtained from AD individuals [55].

## 5. Future Directions and Consideration in Using Injection of A*β* as an Animal Model of AD

Neuroinflammation is a critical component of Alzheimer's disease brain [5, 56, 57]. Animal models using injected A*β* as a stimulus for induction of inflammatory reactivity will have utility in characterization of processes contributing to neurodegeneration in disease. The results described above using the A*β*_1-42_-injected rat model represent correlated data between extents of microgliosis and viability of neurons. As such, the findings are not readily interpretable as to inflammatory reactivity as a contributing causative process for neurodegeneration. To examine the latter process in detail, studies are required which are designed to examine time-dependent changes in microglial responses and neuronal viability over long-term durations following intrahippocampal peptide injection. Such experiments will be useful to determine if microgliosis precedes neuronal loss, examining the mechanisms involved which link inflammatory reactivity with neuron viability and the nature of the neurodegenerative processes. In addition, AD animal models including intrahippocampal injection of peptide and numerous transgenic mouse models have not been extensively studied for abnormalities in synaptic function. However, perturbations in synaptic transmission could constitute an early and sensitive measure of neuronal damage and cognitive impairment in AD brain.

It should be emphasized that the intrahippocampal injection of A*β*_1-42_ represents an AD animal model which amplifies proinflammatory microenvironments and understates anti-inflammatory responses in AD animal brain. This particular animal model is most suitable for investigating effects and mechanisms of actions of a host of compounds which demonstrate anti-inflammatory activity. As noted above, studies of inflammatory responses in peptide-injected rat hippocampus offer some advantages as an alternative to the much more commonly used transgenic mouse models in relation to inflamed human brain.

Another noteworthy point is that although considerable evidence for putative detrimental actions of microglial reactivity is available [2], activation of microglial cells in AD can have positive effects. For example, increased levels of a number of anti-inflammatory factors such as TGF-*β*1 can be produced by activated glial cells in transgenic AD mice and in AD brain [58, 59]. The beneficial responses of activated microglia in disease have been considered [3, 5, 60–62]. Although chronic inflammation may tilt the balance towards proinflammatory reactivity in AD brain, activation of microglia can lead to functional cell responses which confer neuroprotection.

## 6. Conclusions

In summary, animal models using injection of peptide into animal brain offer alternative approaches to the more thoroughly studied transgenic mouse models. Peptide injection models commonly manifest considerable gliosis which, in some studies, has been linked to neuronal damage. In particular, injection of A*β*_1-42_ peptide into rat hippocampus exacerbates inflammatory reactivity in an AD animal model. The amplified proinflammatory responses in peptide-injected rat brain are associated with neurodegenerative processes with drug inhibition of microglial reactivity conferring partial neuroprotection. Importantly, the peptide-injection model appears to replicate the changes in cellular properties and brain microenvironments evident in inflamed brain in the progression of AD pathology.
